# sCD163 levels as a biomarker of disease severity in leprosy and visceral leishmaniasis

**DOI:** 10.1371/journal.pntd.0005486

**Published:** 2017-03-29

**Authors:** Ricardo Luís Louzada Silva, Marcio B. Santos, Priscila L. S. Almeida, Thayse S. Barros, Lucas Magalhães, Rodrigo A. Cazzaniga, Patrícia R. M. Souza, Nívea F. Luz, Jaqueline França-Costa, Valeria M. Borges, Djalma S. Lima-Junior, Michael W. Lipscomb, Malcolm S. Duthie, Steven G. Reed, Roque Pacheco Almeida, Amélia Ribeiro Jesus

**Affiliations:** 1 Laboratório de Biologia Molecular–Hospital Universitário–Universidade Federal de Sergipe–Aracaju—Brazil; 2 Departamento de Educação em Saúde de Lagarto–Universidade Federal de Sergipe–Lagarto–Brazil; 3 Centro de Pesquisas Gonçalo Moniz (CPqGM), Fundação Oswaldo Cruz (FIOCRUZ), Salvador, Brazil; 4 Departamento de Bioquímica e Imunologia–Faculdade de Medicina de Ribeirão Preto–Universidade de São Paulo–Ribeirão Preto–Brazil; 5 Department of Biology, Howard University, Washington–DC–United States of America; 6 Infectious Diseases Research Institute (IDRI)–Seattle–WA–United States of America; Hospital Universitário Professor Edgard Santos, BRAZIL

## Abstract

**Background:**

CD163, receptor for the haptoglobin–hemoglobin complex, is expressed on monocytes/macrophages and neutrophils. A soluble form of CD163 (sCD163) has been associated with the M2 macrophage phenotype, and M2 macrophages have been shown to down-modulate inflammatory responses. In particular, previous studies have shown that M2 is closely associated with the most severe clinical presentation of leprosy (i.e. lepromatous leprosy (LL)), as well as tuberculosis. We hypothesized that sCD163 correlates with severity of diseases caused by intracellular pathogens.

**Methodology/Principal findings:**

To assess this hypothesis, sCD163 levels were measured in the serum of leprosy and visceral leishmaniasis (VL) patients stratified by severity of the clinical presentation. sCD163 levels were significantly higher in patients with these diseases than those observed in healthy control individuals. Further analyses on infection and disease status of leprosy and VL patients revealed a clear association of sCD163 levels with clinical parameters of disease severity. *In vitro* culture assays revealed that *Leishmania* infection induced CD163 expression on the surface of both monocyte/macrophages and neutrophils, suggesting these cells as possible sources of sCD163. FACS analyses shows that the cells expressing CD163 produces both TNF-α and IL-4.

**Conclusions/Significance:**

Taken together, our results reveal sCD163 as a potential biomarker of severity of diseases caused by intracellular pathogens *M*. *leprae* and *Leishmania* spp. and have a modulatory role, with a mix of an inflammatory property induced by TNF-α release, but that potentially induces an anti-inflammatory T cell response, related to IL-4 release.

## Author summary

Visceral leishmaniasis (VL) is a systemic, and most severe form of leishmaniasis. Soluble CD163 (sCD163) levels can serve as biomarker for disease severity in several inflammatory disorders. However, no linkage has been reported for its relationship with *Leishmania* infections. We now demonstrate, for the first time, that sCD163 is increased in VL patients, and its presence is directly correlated to clinical parameters of disease severity. *In vitro* infection of monocyte-derived macrophages and neutrophils with *L*. *infantum* and *L*. *amazonensis* induces, while BCG reduce the expression of CD163 on macrophage surface Furthermore, presence of sCD163 is reduced during clinical improvements. Taken together, results reveal an important role for sCD163 in immune modulation during disease progression, and suggest a potential role as biomarker for determining disease severity and clearance.

## Introduction

CD163 is a member of the scavenger receptor cysteine-rich family [1]. CD163 binds to hemoglobin (Hb) and haptoglobin (Hp) complex [2] and helps to coordinate the receptor-mediated endocytosis by phagocytes [3] to be processed by hemeoxygenase-1 (HO-1) [4,5]. CD163, largely expressed on monocytes/macrophages and neutrophils [6,7], has several roles as an extracellular sensor for bacteria and modulator of immunological responses [8]. Alternatively activated macrophages, or M2, have anti-inflammatory and tissue repair properties and have been described to express CD163 [9,10]. CD163 can be shed from the macrophage surface in response to inflammatory stimuli [3], and can then be found as a soluble form (i.e. sCD163) [7,11]. Macrophages expressing CD163 have been described in lepromatous leprosy (LL), the most severe presentation of the infectious disease caused by *Mycobacterium leprae*, with CD163 facilitating bacterial survival by providing a source of iron for mycobacterial survival as well as triggering IL-10 production [7]. sCD163 has been identified as an indicator of disease severity in several inflammatory and infectious diseases [7,9,12–15].

Several prognostic and severity markers have been described in visceral leishmaniasis (VL) patients, including mucosal bleeding, jaundice, dyspnea, suspected or confirmed bacterial infections, neutrophil count <500/mm^3^ and platelet count <50,000/mm^3^ [16]. In addition, dos Santos et al (2016) reported that IL-6, IL-27 and sCD14 can serve as useful biomarkers for severity of VL [17], and that IL-6 levels greater than 200 pg/ml were strongly associated with death. Interestingly, CD163 is upregulated by interleukin-6 (IL-6) and IL-10 [5,18,19], two cytokines described to be high in VL patients [17,20–22], but linkage of CD163 has not yet been reported for *Leishmania* infection. We therefore hypothesized that circulating sCD163 levels would correlate with severity of diseases caused by intracellular pathogens, such as leprosy and VL. We measured sCD163 levels in the sera of leprosy and VL patients to determine whether association could be made with severity of these diseases. Moreover, to determine if CD163 was simply indicative of disease state or might be involved in disease pathogenesis, we performed *in vitro* experiments to determine the impact of *Leishmania* infection on CD163 expression on macrophages and neutrophils.

## Methods

### Ethical considerations

The Ethics and Research Committee of the Federal University of Sergipe approved this study (CAAE 0151.0.107.000–07 and CAAE 0152.0.107.000–07) and all recruits, or their legal guardians, willfully consented. All recruits provided written informed consent (as outlined in the PLOS consent form) to publication of their case details.

### Patients and control individuals

Leprosy patients and their pertinent controls were enrolled at the Leprosy Clinic from the University Hospital, Federal University of Sergipe, in Sergipe State, Brazil (HU-UFS). They were classified according to the Madrid (1953) criteria of clinical forms: Indeterminate Leprosy (IL, n = 9), Tuberculoid Leprosy (TT, n = 14), Borderline Leprosy (BL, n = 14) and Lepromatous Leprosy (LL, n = 10) [23]. The inclusion criteria were a diagnosis of leprosy confirmed by clinical aspects of the lesions and either positive bacilloscopy or histopathological confirmation in skin biopsies. Exclusion criteria were having other conditions (pregnancy) or diseases (HIV, HTLV-1, Diabetes) that interfere in the immune response or in the clinical outcome of leprosy. After collection of blood and tissue samples, patients were treated following the standard multidrug therapy (MDT), according to the Brazilian Ministry of Health and World Health Organization guidelines. Sera of household contacts of patients (Contacts; n = 23) were used as controls. Contacts were individuals who lived in direct and prolonged contact with the leprosy patients and who submitted to careful dermatological exam to exclude the presentation of leprosy at the time of recruitment. As a group at elevated risk of *M*. *leprae* infection, however, we could not formally exclude the possibility that these contacts are infected or may become ill in the future.

Clinical data and sera for VL patients, and their pertinent controls, were obtained from a database of the VL Reference Center at HU-UFS, Sergipe, Brazil. VL patients were divided into five groups; (1) before treatment (D0-Classic, n = 33), (2) 30 days after diagnosis with VL (after treatment) (D30, n = 19), (3) severe VL at day 0 (D0-SVL, n = 13), (4) asymptomatic (delayed type-hypersensitivity (DTH)-positive, n = 11) and (5) non-endemic health controls (HC, n = 8). DTH positive individuals are people who live with the patients and are responsive as measured by the DTH skin test positive for *Leishmania* soluble antigen, but do not have clinical symptoms of the disease. Patients were classified as having severe VL based on clinical features that included platelet counts <50,000/mm^3^, bleeding, bacterial infections, neutrophil counts <500/mm^3^, dyspnea and jaundice, as described by Sampaio et al. [16]. The inclusion criteria were VL diagnosis confirmed by direct observation of *Leishmania* in bone marrow aspirates or positive culture in NNN media (Sigma-Aldrich), or a positive response in the rK39 serological test (KalazarDetect Rapid Test: InBios International Inc). Patients were submitted to standard VL treatment with Antimonial (Sb^v^) [24]. Exclusion criteria were having other conditions (pregnancy) or diseases (HIV, HTLV-1, Diabetes) that interfere in the immune response or in the clinical outcome of VL.

### Measurement of biomarkers in serum samples

Blood was collected from all volunteers and serum prepared. All sera samples were stored at -80°C until analyses. sCD163 quantification were performed at the same time for all sera samples by ELISA kit according to the manufacturer’s instructions (R&D Systems). Haptoglobin was measured using a kit from GenWay, Heme-oxygenase I was measured using a kit from Assay Designs and Arginase-1 was measured using a kit from Hycult Biotech, following the manufacturer’s instructions.

### Cell isolation and culture

Monocytes were isolated from peripheral blood and plated in 24 well-plates at 5x10^5^ cells/well. Differentiation of macrophages were performed as previously described by de Oliveira et al [25]. Neutrophils were isolated from peripheral blood samples of healthy donors (with EDTA as anticoagulant) using PolimorphPrep reagent, according to the manufacturer’s instructions (Axis-Shield). The cells were washed with PBS prior to seeding into 96 well plates at a concentration of 10^6^ neutrophils/well in RPMI 1640 supplemented with 10% FBS.

### *In vitro Leishmania* spp. and BCG infection

*L*. *(L*.*) amazonensis* strain (MHOM/BR/73M2269) [26] that constitutively expresses GFP and two different *L*. *infantum* isolates from VL patients (MHOM/BR/2009/LVHSE17 as isolate 1 and MHOM/BR/2010/LVHSE49 as isolate 2) were used [27]. *L*. *amazonensis-*GFP was constructed by incorporation of the GFP gene into 18s ribosomal RNA by homologous recombination using pSSU vector, as described by Misslitz et al. (2000) [28]. *L*. *infantum* isolates were stained with CellTracker Violet BMQC dye (Thermo Fisher) as previously described [29]. The parasites were cultured axenically in Schneider's *Drosophila* medium (Thermo Fisher) plus 10% FBS prior to infection of cells. Infection of human macrophages was performed at a ratio of either 10 stationary-phase *L*. *amazonensis-*GFP promastigotes or 5 *L*. *infantum* per macrophage. Extracellular parasites were removed 2 hours later by washing. After 24h, the cells were then stained and subjected to flow cytometry. Neutrophils were infected at a ratio of 5 *L*. *amazonensis* parasites per neutrophil for 3h prior to staining and flow cytometry. Infection with *Mycobaterium*. *bovis* BCG (Fundação Ataulpho de Paiva) was performed at a ratio of 2 mycobacteria per macrophage. BCG were also stained with CellTracker Violet BMQC dye, following the same protocol used for *Leishmania*.

### Flow cytometry

Cells were washed with PBS and incubated with fluorescently-labeled antibodies according to the manufacturer’s instructions (BD Biosciences, USA). Cells were incubated with anti-CD209-BV421 (cat. 564127), anti-CD163-PE (cat. 556018), anti-CD86-BV510 (cat. 563461) and/or anti-CD40-APC (cat. 555591). To identify neutrophils, cells were incubated with anti-CD15-BV450 (cat. 561584) and anti-CD163-PE. To assess cytokine expression, cells were incubated with BD Cytofix/Cytoperm reagent (cat. 554722), anti-TNF-alpha-PerCP-Cy5.5 (cat. 560679), anti-IL10-APC (cat. 554707), anti-IL-4-PerCP-Cy5.5 (cat. 561234) and anti-IL-12-APC (cat. 554576). Cells were fixed with 4% paraformaldehyde prior to acquisition on a FACS CANTO II (BD Biosciences) and data was analyzed using FlowJo v10.0 software (Tree Star). The gating strategy was to first set a gate in the FSC/SSC in the regions compatible with either macrophages morphology (macrophage surface phenotype and cytokine analysis) or neutrophil morphology. For surface phenotype analysis, the second step was to distinguish infected from uninfected cells using FSC vs GFP or Celltracker dot plots. The third step was to set a positive gate for each marker according to the florescence of each label versus FSC.

### Statistical analyses

Statistical analyses were performed using Windows GraphPad Prism version 5.0 (GraphPad Software).Results are expressed as mean ± standard deviation (SD). D’Agostinho-Pearson normality test was performed to establish if the data had a normal distribution. Differences between two groups were determined by Mann-Whitney test (sCD163 analysis). Parametric t paired test was used in before/after treatment analysis. Friedman paired test with Dunn´s post test (Surface Phenotype and Median of Fluorescence Intensity analysis) and Wilcoxon paired test (Intracellular cytokine analysis) were used for non-parametric paired analyses. Correlation analysis was performed using Spearman correlation test. A p-value ≤ 0.05 was considered significant.

## Results

### sCD163 is elevated in the serum of lepromatous leprosy patients

**Fig 1:**
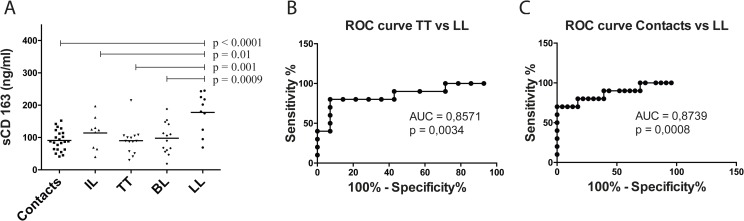
sCD163 is elevated in the serum of lepromatous leprosy patients. (A) Sera of leprosy patients with various clinical presentations were collected and sCD163 concentrations measured by ELISA. Household contacts without symptoms or signs of leprosy (Contacts) were used as a control group. The mean ± SD sCD163 levels in patients with indeterminate leprosy (IL) (n = 9; 114 ± 49,57 ng/mL), true tuberculoid leprosy (TT) (n = 14; 90,29 ± 44,06 ng/mL), borderline leprosy (BL) (n = 14; 97,71 ± 47,97 ng/mL) and lepromatous leprosy (LL) (n = 10; 177,6 ± 62,18 ng/mL), as well as contacts (n = 23; 90,78 ± 31,55 ng/mL) were compared by Mann-Whitney test. ROC curves comparing sCD163 concentrations from TT versus LL (B) and Contacts versus LL patients (C) were constructed and are shown.

Patients with the most heavily infected and severe manifestation of leprosy, LL, had higher serum sCD163 levels than both households contacts of patients (contacts) (p<0.001, Mann-Whitney test) and patients presenting with tuberculoid leprosy (TT) (p = 0,001, Mann-Whitney test), indeterminate leprosy (IL) (p = 0.01, Mann-Whitney test) or borderline leprosy (BL) (p = 0.0009, Mann-Whitney test) (Fig 1A). Receiver Operating Characteristic (ROC) curves were constructed and area under the curve (AUC) analysis highlighted the utility of sCD163 levels for distinguishing between LL and either TT (AUC = 0.8571, 95% confidence interval (CI) [0.69–1.02], p = 0.0034) or contacts (AUC = 0.8439, 95% CI [0.72–1.02], p = 0.0008) (Fig 1B and 1C). In contrast, levels of haptoglobin, heme-oxygenase-1 and arginase-1 were not different between the groups (p>0.05 for all comparisons, Student t test or Mann-Whitney test). The mean ± SD of haptoglobin levels in these groups were: LL (46.4 ± 21.00), TT (45.7 ± 39.50) and Contacts (47.3 ± 31.78); of heme-oxygenase-1 were: LL (0.6 ± 0.34), TT (0.4 ± 0.16) and Contacts (0.4 ± 0.24); and arginase-1 were: LL (16.9 ± 11.04), TT (10.6 ± 4.60) and Contacts (15.6 ± 9.24).

### sCD163 levels correlate with severity of visceral leishmaniasis (VL)

**Fig 2:**
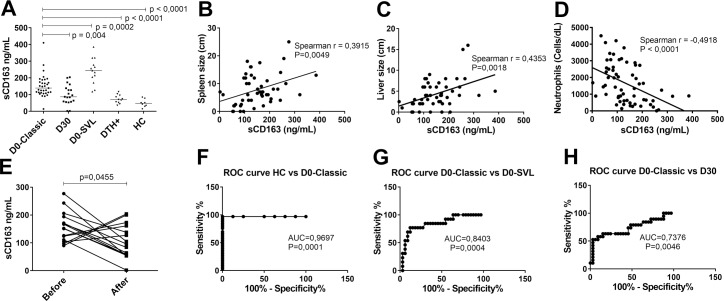
sCD163 levels correlate with severity of VL. (A) sCD163 levels were measured in sera of VL patients of different clinical status. The mean ± SD sCD163 levels of patients with classical VL at D0 (D0-Classic, n = 33) (152,1 ± 67,86 ng/mL), D30 (n = 19) (98,79 ± 58,58 ng/mL) and of patients of severe VL at D0 (D0-SVL) (n = 13) (241,5 ± 76,88 ng/mL) were compared by Mann-Whitney test. Sera from *Leishmania*-infected individuals without symptoms or signs of VL (DTH+, n = 11, 72,55 ± 25,68 ng/mL) and healthy individuals from non-endemic regions (HC, n = 8, 49,0 ± 23,71 ng/mL) were included as control groups. Spearman correlation analyses between sCD163 concentrations were performed versus (B) spleen size, (C) liver size and (D) neutrophil count. (E) Paired analysis of sCD163 levels of VL patients before and after treatment (n = 15, p = 0.0455, paired t test). ROC curves of sCD163 concentration comparing HC versus (F) D0-Classic, (G) D0-Classic versus D30 and (H) D0 versus D0-SVL group.

We detected high serum levels of sCD163 in VL patients compared to healthy individuals from non-endemic regions (HC) and *Leishmania*-infected but healthy controls (delayed-type hypersensitivity-positive; DTH+) (p<0.0001, Mann-Whitney test) (Fig 2A) [17]. Fig 2F shows the distinction between control group (HC) versus D0-classic patients (AUC = 0,9697, CI [0,9112–1,2], p = 0,0001), by ROC curve, reiterating the value of sCD163 as a biomarker of disease. The highest sCD163 levels were detected in patients classified as presenting with severe VL (D0-SVL) (p<0.004, Mann-Whitney test) (Fig 2A). ROC analysis of D0-classic versus D0-SVL patients (AUC = 0,8403, CI [0,7101–0,97], p = 0,0004) (Fig 2G) provides further support for the use of sCD163 in determining disease severity and clinical improvement, respectively. A direct correlation was observed between serum sCD163 levels and both spleen size (Spearman r = 0.3915) (Fig 2B) and liver size (r = 0.4353) (Fig 2C), while an inverse correlation was observed between sCD163 concentration and neutrophil counts of VL patients (r = -0.4918) (Fig 2D). These measures represent standard clinical parameters used for determining VL severity [17], and our results therefore indicate the potential of using serum sCD163 levels as an indicator of VL severity.

### sCD163 levels of visceral leishmaniasis (VL) patients decline upon treatment

Having demonstrated the linkage of sCD163 levels with severity of VL, we speculated that levels would be reduced as disease resolved upon treatment. Accordingly, paired analysis of samples collected before and after treatment (D0-Classic and D30, respectively) demonstrated a reduction of sCD163 serum levels in 10 of 15 patients after the completion of treatment (Fig 2E; p = 0.0455, paired t test). ROC analysis of D0-classic versus D30 group (AUC = 0,7376, CI [0,5832–0,89], p = 0,0046) (Fig 2H) provides further support for the use of sCD163 in monitoring clinical improvement. These data support our hypothesis that the decrease of sCD163 levels can be used as an indicator of treatment success.

In contrast to the sCD163 data, significant differences were not observed between these groups in terms of haptoglobin, HO-1 or arginase-1 levels (p>0.05 for all comparisons, Student t test or Mann-Whitney test). The mean ± SD of haptoglobin levels in D0-Classic (25.8 ± 29.82), D30 (10.2 ± 6.46), D0-SVL (11.6 ± 9.72) and DTH+ (14.5 ± 6.75); heme-oxygenase-1 in D0-Classic (1.6 ± 2.13), D30 (0.1 ± 0.03), D0-SVL (2.1 ± 3.98) and DTH+ (0.2 ± 0.003); and arginase-1 in D0-Classic (7.2 ± 6.38), D30 (7.1 ± 6.23), D0-SVL (12.2 ± 14.33) and DTH+ (3.5 ± 3.09).

### *Leishmania* infection and macrophage CD163 expression

**Fig 3:**
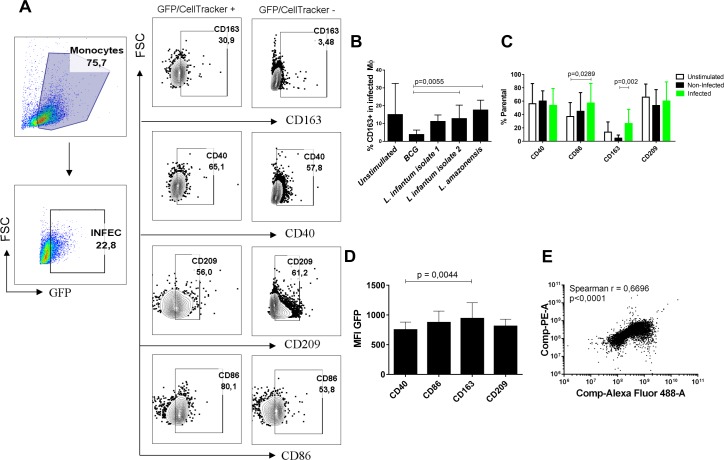
CD163 expression is induced by *Leishmania* infection of monocyte/macrophages. (A) Gating strategy for analysis of monocyte/macrophage phenotypes. PBMC were collected from healthy donors and the adherent cells cultured for 5 days in RPMI 1640 plus 20% FBS. The cells were infected with *L*. *amazonensis-*GFP (10 parasites: 1 macrophage), two isolates of *L*. *infantum* CellTracker stained (5:1) or BCG CellTracker stained (2:1), and incubated with antibodies specific for macrophage surface molecules prior to analysis by flow cytometry. (B) Surface CD163 expression was quantified after 24h in infected, GFP+ cells (in duplicate, n = 5 experiments). (C) The macrophages were analyzed by FlowJo software (in duplicate, n = 6 experiments). Green and black bars represent, respectively, the infected, GFP+ and uninfected, GFP- cells after 24h of exposure to *L*. *amazonensis* GFP-expressing strain. White bars are the non-exposed group (unstimulated). The mean ± SD parental percentage of the cell populations (B and C) were compared by Friedman paired test with Dunn’s post test. (C) MFI analysis of GFP in the different populations according to the surface phenotype, 24h after infection (n = 7 experiments in duplicate). The mean ± SD MFI of each population was compared by Friedman paired test with Dunn’s post test. (D) Spearman correlation analysis between CD163-PE and *Leishmania-*GFP fluorescence for each cell of the analysis (one representative of 7 experiments in duplicate).

To assess the impact of infection on CD163 we infected cells in vitro with various *Leishmania* species. CD163, CD40, CD209 and CD86 expression were evaluated in macrophages incubated with a *Leishmania amazonensis* strain expressing GFP, two *L*. *infantum* isolates stained with Violet Celltracker or BCG also stained with Celltracker (Fig 3A). While *L*. *infantum* and *L*. *amazonensis* infection induces CD163 expression in macrophage surface, BCG infection did not (Fig 3B). Macrophages exposed to *L*. *amazonensis* for 24 hours yielded two cells subpopulations [30], with GFP+ cells considered infected while GFP- cells were assumed to be non-infected. The infected cells had a higher percentage of CD86+ (57.11%) and CD163+ (33.6%) cells compared to both non-infected (CD86 44,78%, CD163 3,22%) and unstimulated cells (CD86 36,93%, CD163 12,62%) (Fig 3C). The median fluorescence intensity (MFI) of GFP was evaluated in CD40, CD86, CD209 and/or CD163 positive populations to assess the relationship of parasite load with these surface molecules. CD163+ cells showed higher infection (MFI = 913.167) than CD40+CD163- (MFI = 715.143) cells (p = 0.0044, Friedman paired test) (Fig 3D). A direct correlation was observed between the CD163 expression levels and *Leishmania* infection (GFP) levels (r = 0,67, p<0,0001) (Fig 3E). These results support the hypothesis that highly infected macrophages could be a source of sCD163.

### CD163+ cells are TNF-α and IL-4 producers

**Fig 4:**
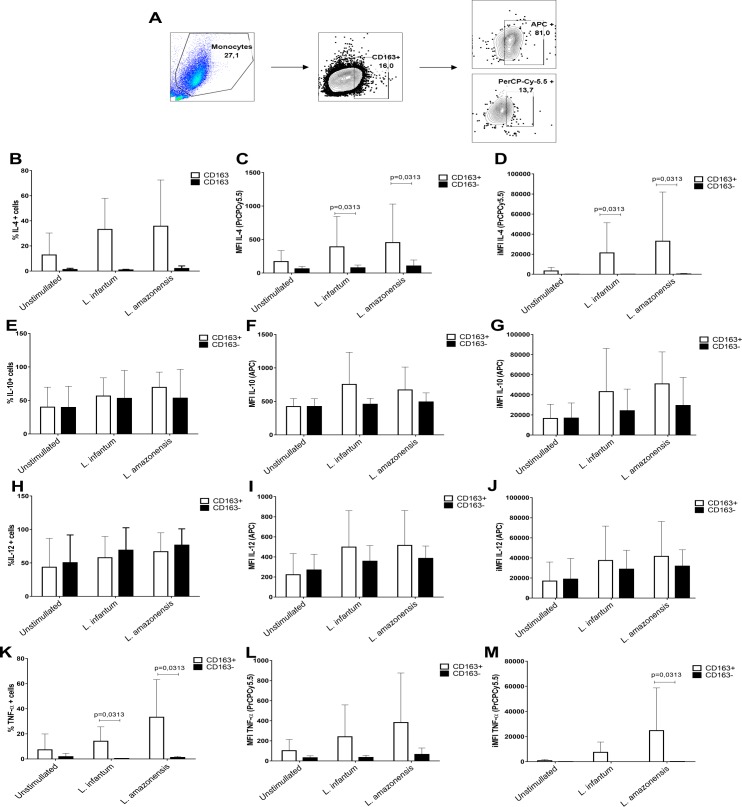
Variable cytokine production by CD163+ and CD163- monocyte/macrophages. (A) Gating strategy for analysis of the cytokine profiles of CD163+ and CD163- monocyte/macrophages. PBMC were collected from healthy donors and the adherent cells cultured for 5 days in RPMI 1640 plus 20% FBS. The cells were incubated with *L*. *amazonensis* strain (10 parasites: 1 macrophage) and *L*. *infantum-*isolate 1 (5:1), and incubated with antibodies specific for intracellular cytokines, prior to analysis by flow cytometry (n = 6 experiments, in duplicate). (B) Frequency of IL-4+ cells, (C) MFI of IL-4-PerCPCy5.5, (D) iMFI of IL-4 analysis, (E) Frequency of IL-10+ cells, (F) MFI of IL-10-APC, (G) iMFI of IL-10 analysis, (H) Frequency of IL-12+ cells, (I) MFI of IL-12-APC, (J) iMFI of IL-12 analysis, (K) Frequency of TNF-α+ cells, (L) MFI of TNF-α-PerCPCy5.5, (M) iMFI of TNF-α analysis.

To assess the effector function of CD163 expressing macrophages and its implications in the regulation of the immune response, flow cytometry was performed to evaluate the cytokine profile of these cells. A greater frequency of cells expressing TNF-α and IL-4 was detected in the CD163+ population (MFI and integrated MFI; Fig 4B–4D and Fig 4K–4M). We did not observe any differences in IL-12 and IL-10 expression between CD163+ and CD163- cells (Fig 4E–4J).

### *Leishmania* infection induces CD163 expression in neutrophils

**Fig 5:**
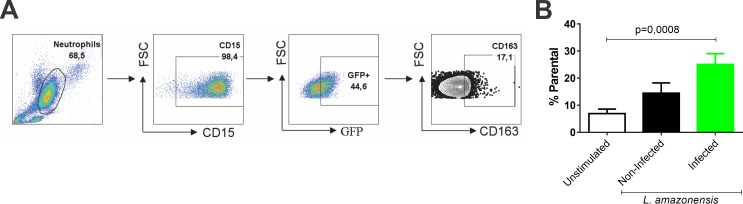
CD163 expression is induced by *Leishmania* infection of neutrophils. (A) Gating strategy for the analysis of neutrophil phenotypes. Neutrophils were purified from healthy donors and infected with GFP-expressing *L*. *amazonensis* (5 parasites: 1 neutrophil) in RPMI 1640 plus 10% FBS. (B) 3h after infection, neutrophils were characterized by flow cytometry and data analyzed by FlowJo software (in duplicate, n = 5 experiments). Green and black bars represent, respectively, the GFP positive and negative cells. The white bars represent non-exposed group (unstimulated). The mean ± SD of parental percentage of GFP+, GFP- and non-exposed cells were compared by Friedman paired test with Dunn’s post test.

To identify if infected neutrophils also expressed CD163, neutrophils from healthy individuals were exposed to *L*. *amazonensis* (Fig 5A). Flow cytometry reveals a higher percentage of CD15+CD163+ cells within the infected, GFP+ population (25.08%) relative to the uninfected, GFP- (14.84%) and unstimulated control populations (6.90%) (p = 0.0008, Friedman paired test) (Fig 5B). These findings parallel those obtained with macrophages. Taken together, our *in vitro* infection data suggest that both macrophages and neutrophils are sources of sCD163 during *Leishmania* infection and that the quantity of CD163 is directly correlated with infection level.

## Discussion

CD163 is a scavenger receptor that has previously been identified as an indicator of disease severity in several inflammatory and infectious diseases [7,9,12–15]. Consistent with this, our data reveal a strong correlation of serum sCD163 levels with the severest clinical presentations of leprosy and, for the first time, VL. Moreover, we observed that *Leishmania* infection induces CD163 expression on monocyte/macrophages and neutrophils, suggesting that these cells might be a source of sCD163 during VL. The infected CD163+ macrophages preferentially produced TNF-α and IL-4. Collectively, our data indicate the induction of CD163 expression during infection with *M*. *leprae* and *Leishmania* species, likely modulating the immune response to permit high levels of infection and the most severe clinical presentations of these diseases.

Our data examining leprosy patients corroborate the previously reported association of serum sCD163 with the lepromatous leprosy (LL) presentation [7]. Moreover, our study extends and refines the previous data by showing that measurement of serum sCD163 can distinguish LL from the other clinical forms of leprosy, including intermediate forms of leprosy (IL and BL). Moura et al (2012) demonstrated also the presence of CD163+ macrophages in lesions of LL patients and therefore attributed the association of the serum levels of sCD163 and severity of leprosy to the differentiation of macrophages to the M2 phenotype [7]. Sousa et al (2016) found a direct correlation between CD163 expression and a variety of inflammatory cytokines in lesions of LL patients, including arginase-1 enzyme expression which is characteristic of M2 macrophages [31]. Interestingly, we did not find differences in the levels of haptoglobin and the enzyme that degrades the hemoglobin-haptoglobin complex (Hb-Hp), heme-oxygenase-1 (HO-1) and arginase-1 among the groups, suggesting that sCD163 levels are a more robust physiologic alteration.

We also detected higher levels of sCD163 in the serum of VL patients, with these levels correlating with multiple clinical parameters of disease severity. The direct correlation of serum sCD163 concentration with liver, spleen size and an inverse correlation with neutrophil counts supports the use of sCD163 as a surrogate indicator of disease severity in VL patients. In addition, a decrease in the levels of sCD163 was observed between the start and end of treatment (D0 versus D30), suggesting that sCD163 measurements could be used to monitor response to treatment. Declines were not observed in all patients, however, with 5 of the 15 patients presenting with similar or even higher levels of sCD163 at the end of treatment. At D30 the VL patients are still recovering from various symptoms of this disease, and a longer follow-up may be beneficial and further studies are required to more strenuously determine the utility of this biomarker in case management.

Based on the observations from patient samples, we evaluated how CD163 expression is induced and what the likely cellular sources of this molecule are. We observed that infection by *Leishmania* induced CD163 expression on the surface of both macrophages and neutrophils, identifying these cells as potential sources of the sCD163 detected in serum. This was true for two *Leishmania* species (observed with *L*. *amazonensis*—GFP and two isolates of *L*. *infantum*) and this is the first study demonstrating that *Leishmania* parasites can induce this macrophage phenotype. In leprosy, a similar relationship has previously been suggested by the observation in lesion biopsies of macrophages expressing CD163 that are heavily infected with *M*. *leprae* [31]. Moreover, Moura et al (2012) showed CD163 expression on monocytes can be induced by *M*. *leprae* infection *in vitro* [7].

During *M*. *leprae* infection co-expression of CD209 and CD163 is indicative of a permissive and phagocytic programming of macrophages characteristic of heavily infected lesions [32]. Interestingly, BCG is a potent pro-inflammatory stimulus with as a previously demonstrated macrophage polarization to a M1-like phenotype, [33] and our data shows that BCG down regulates CD163 expression. Thus, we observed striking differences in the response to pathogenic *M*. *leprae* and nonpathogenic *M*. *bovis* BCG. In our model *Leishmania* infection did not lead to increases in CD209 positive cells with uninfected and infected populations having the same frequency of CD209+ macrophages. Under the *in vitro* conditions we used, only CD163+ cells was more frequent in the infected than in the uninfected population. CD209 is a marker expressed in earlier stages of activation and it is therefore possible that infection kinetics may have an impact. Regardless, these data denote some differences in the responses to these intracellular pathogens.

Flow cytometry analysis found that not only is the CD163+ population more prone to infection with *Leishmania* parasites but produces more IL-4 and TNF-α than CD163- macrophages. No differences were observed in IL-12 or IL-10 production between these phenotypes. Saha, et al. (2016) also found in hepatitis C infection that macrophages expressing surface markers of M2 can produce both anti- and pro-inflammatory cytokines [34]. In the complex evolution of VL, these responses might interfere in instructing T cells and inhibit the killing of these intracellular pathogens. Moura et al. (2012) has also suggested that these macrophages are characteristic of M2 subtype that is more permissive for *M*. *leprae* infection [7]. In addition to macrophages, neutrophils infected with *L*. *amazonensis* also express CD163 and may be another source for sCD163 in the serum of VL patients. Groselj-Grenc et al (2008) found expression of CD163 on neutrophils in systemic inflammatory response syndrome [6] and an immunomodulatory role of polymorphonuclear leukocytes has been described during the early phase of *L*. *major* infection [35]. We suggest that CD163 may be related to differentiation of these neutrophils toward an anti-inflammatory N2 subtype, such as has recently described [36,37]. Further studies are needed to confirm the presence of CD163 positive neutrophils in VL patients during active disease.

## Conclusion

In conclusion, our data indicate the potential use of serum levels of sCD163 in indicating severity of diseases caused by intracellular pathogens. Our results corroborate and expand previous findings for leprosy and, for the first time, demonstrate high levels of sCD163 correlate with severe clinical symptom observed in VL patients. This study also suggests that infected macrophages and neutrophils are possible sources of sCD163, and show that both *L*. *amazonensis* and *L*. *infantum* can polarize macrophages to produce both pro- (and anti- inflammatory cytokines (TNF-α and IL-4, respectively). This would appear to favor parasite multiplication and exacerbate clinical presentation.
